# Anti-Inflammatory Effect of Methylpenicinoline from a Marine Isolate of *Penicillium* sp. (SF-5995): Inhibition of NF-κB and MAPK Pathways in Lipopolysaccharide-Induced RAW264.7 Macrophages and BV2 Microglia

**DOI:** 10.3390/molecules191118073

**Published:** 2014-11-05

**Authors:** Dong-Cheol Kim, Hee-Suk Lee, Wonmin Ko, Dong-Sung Lee, Jae Hak Sohn, Joung Han Yim, Youn-Chul Kim, Hyuncheol Oh

**Affiliations:** 1 Institute of Pharmaceutical Research and Development, College of Pharmacy, Wonkwang University, Iksan 570-749, Korea; E-Mails: kimman07@hanmail.net (D.-C.K.); cutehs22@naver.com (H.-S.L.); rabis815@naver.com (W.K.); 2 Inha Research Institute for Medical Sciences, Inha University School of Medicine, Incheon 400-712, Korea; E-Mail: dongsunglee@inha.ac.kr; 3 College of Medical and Life Sciences, Silla University, Busan 617-736, Korea; E-Mail: jhsohn@silla.ac.kr; 4 Korea Polar Research Institute, KORDI, 7-50 Songdo-dong, Yeonsu-gu, Incheon 406-840, Korea; E-Mail: jhyim@kopri.re.kr

**Keywords:** methylpenicinoline, *Penicillium* sp., marine fungus, anti-inflammation, nuclear factor-kappa B (NF-κB), mitogen-activated protein kinase (MAPK)

## Abstract

In the course of a search for anti-inflammatory metabolites from marine-derived fungi, methylpenicinoline (**1**) was isolated from a marine isolate of *Penicillin* sp. Compound **1** inhibited lipopolysaccharide (LPS)-stimulated nitric oxide (NO) production by suppressing the expression of inducible NO synthase (iNOS) in RAW264.7 macrophages and BV2 microglia. It also attenuated prostaglandin E_2_ (PGE_2_) production by suppressing cyclooxygenase-2 (COX-2) expression in a concentration-dependent manner (from 10 μM to 80 μM) without affecting cell viability. In addition, compound **1** reduced the production of the pro-inflammatory cytokine interleukin-1β (IL-1β). In a further study designed to elucidate the mechanism of its anti-inflammatory effects, compound **1** was shown to block nuclear factor-kappa B (NF-κB) activation in LPS-induced RAW264.7 macrophages and BV2 microglia by inhibiting the phosphorylation of inhibitor kappa B-α (IκB-α), thereby suppressing the nuclear translocation of NF-κB dimers, namely p50 and p65, that are known to be crucial molecules associated with iNOS and COX-2 expression. In addition, compound **1** inhibited the activation of mitogen-activated protein kinase (MAPK) pathways. Taken together, the results suggest that compound **1** might be a valuable therapeutic agent for the treatment of anti-inflammatory and anti-neuroinflammatory diseases.

## 1. Introduction

Inflammation is a temporally and spatially regulated key process of the host defense system. However, an uncontrolled inflammatory reaction can lead to a variety of diseases, including hepatitis, septic shock, arthritis, and neurodegenerative disorders. Macrophages play a central role in inflammation and host defense mechanisms. Upon activation by various intrinsic or extrinsic stimuli, macrophages generate several pro-inflammatory cytokines and mediators, such as tumor necrosis factor (TNF-α), interleukin 1β (IL-1β), nitric oxide (NO), and prostaglandins (PGs). This process is an essential feature of the inflammatory response [1]. This response has been extensively studied in LPS-stimulated RAW264.7 macrophage cells because the cells are very susceptible to LPS stimulation by activation of multiple inflammatory signals [2]. Microglia are commonly regarded as brain macrophages, which are stimulated by various stimuli. Over-activation or persistent activation of microglia plays a role in the pathogenesis of several neurodegenerative diseases, including stroke, Alzheimer’s disease, Parkinson’s disease, multiple sclerosis, and HIV-associated dementia [3]. Thus, the inhibition of pro-inflammatory enzymes and cytokines can be considered as an effective therapeutic access against neurodegenerative diseases. BV2 microglia, of an immortalized murine microglia cell line, are widely used as an *in vitro* model of microglia due to their similarities in morphological and functional features with those of primary microglia [4].

Blockade of the aforementioned inflammatory responses is a target for the development of therapeutic agents [5]. Accordingly, natural products have been investigated as a potential source of novel small molecules that may specifically modulate inflammatory responses [6,7,8].

Because of the unique features of the marine environment, marine fungi are a potential source of diverse novel secondary metabolites [9,10,11,12]. In our ongoing studies on the bioactive secondary metabolites isolated from marine fungi [13,14,15,16], we have conducted the chemical investigation of the extracts obtained from cultures of a marine-derived isolate of *Penicillium* sp. (SF-5995). In this investigation, RAW264.7 macrophages and BV2 cells have been employed as a bioassay system to identify anti-inflammatory metabolite(s). This article describes the isolation of methylpenicinoline (**1**) from extracts of *Penicillium* sp. SF-5995 and its anti-inflammatory and anti-neuroinflammatory effects in RAW264.7 macrophages and BV2 microglia, respectively.

## 2. Results and Discussion

### 2.1. Isolation and Structure Determination of Methylpenicinoline (**1**)

**Figure 1:**
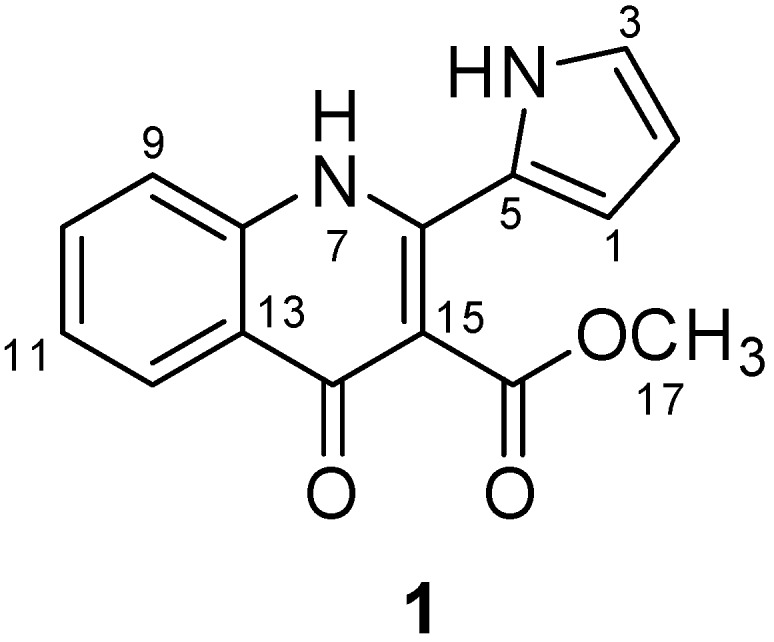
Chemical structure of methylpenicinoline (**1**).

To isolate and identify the anti-inflammatory metabolite(s) from an organic extract of cultures of the marine fungus *Penicillium* sp. SF-5995, bioassay- and ^1^H-NMR-guided fractionation and purification steps were undertaken using C_18_-functionalized silica gel column chromatography and HPLC. This led to the isolation of an unusual pyrrolyl 4-quinoline alkaloid, methylpenicinoline (**1**, Figure 1). The structure of the isolated compound was mainly identified by analysis of various NMR data, coupled with comparison of its spectral data with those in the literature [17].

### 2.2. Effects of Methylpenicinoline (**1**) on the Viability of Mouse-Derived RAW264.7 and BV2 Cells

**Figure 2:**
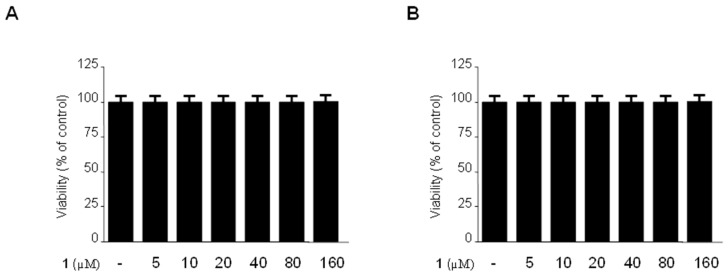
Effects of **1** on cell viability of RAW264.7 macrophages (**A**) and BV2 microglia (**B**). Cells were incubated for 24 h with the indicated concentrations of **1** (5–160 μM). Cell viability was determined as described in Section 3.4. Data are mean ± standard deviation (S.D.) of 3 independent experiments.

To exclude the possibility of direct toxicity of methyl-penicinoline (**1**), the cytotoxicity of **1** against RAW264.7 macrophages and BV2 microglia was assessed by the MTT assay. As shown in Figure 2, cell viability of RAW264.7 macrophages (Figure 2A) and BV2 microglia (Figure 2B) was not significantly affected after incubation with 5–160 µM of **1** for 24 h. Concentrations of **1** below 160 µM were selected for further studies.

### 2.3. Effects of Methylpenicinoline (**1**) on the Production of Pro-Inflammatory Mediators and Cytokines in RAW264.7 Macrophages Stimulated with LPS

**Figure 3:**
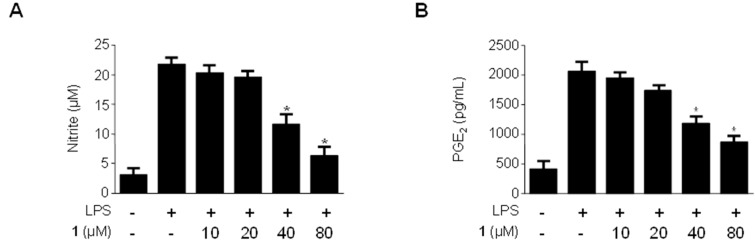

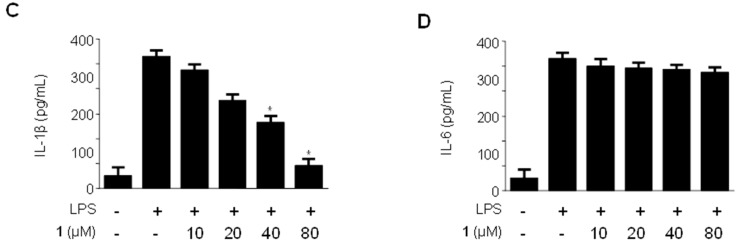
Effects of methylpenicinoline (**1**) on nitrite (**A**); PGE_2_ (**B**); IL-1β (**C**); and IL-6 (**D**) production in RAW264.7 macrophages stimulated with LPS. Cells were pre-treated for 30 min with indicated concentrations of **1**, and then stimulated for 24 h with LPS (500 ng/mL). The concentrations of nitrite (A); PGE_2_ (B); IL-1β (C); and IL-6 (D) were determined as described in Section 3.5. Data represent the mean values of three experiments ± S.D. * *p* < 0.05 compared to the group treated with LPS alone.

NO is a free radical reported to be involved in many physiological and pathological processes. NO is synthesized by the oxidation of L-arginine by nitric oxide synthase [18]. When activated by inflammatory mediators, macrophages are the main producers of NO at inflammatory sites [19,20]. PGE_2_ is another inflammatory mediator generated at inflammatory sites by COX-2, officially named prostaglandin endoperoxide synthase. PGE_2_ is associated with many chronic inflammatory diseases, including cardiovascular diseases, arthritis, inflammatory bowel disease, and chronic gastric ulcers [21,22,23,24]. To evaluate the anti-inflammatory effects of methylpenicinoline (**1**) on LPS-stimulated RAW264.7 macrophages, the concentrations of the pro-inflammatory mediators NO and PGE_2_ were assessed in the presence and absence of compound **1** at non-cytotoxic concentrations ranging from 10–80 µM. RAW264.7 macrophages were pretreated with compound **1** for 30 min, followed by stimulation with LPS (1 µg/mL) for 24 h. As shown in Figure 3, LPS treatment triggered an approximately 10-fold increase in nitrite concentration in the culture media, compared to that of the untreated group. However, pre-treatment of RAW264.7 cells with compound **1** for 30 min decreased the production of NO, with an IC_50_ value of 42 μM (Figure 3A). Under the same conditions, compound 1 also suppressed PGE_2_ production in a concentration-dependent manner, with an IC_50_ value of 39 μM (Figure 3B). Thus, compound **1** suppressed the production of LPS-induced pro-infiammatory mediators. This observation led us to further investigate the effects of compound **1** on the production of the LPS-induced pro-inflammatory cytokines IL-6 and IL-1β. As shown in Figure 3C, compound **1** decreased IL-1β production in a concentration-dependent manner, with an IC_50_ value of 36 μM, while showing very weak or no effect on the production of IL-6 (Figure 3D).

### 2.4. Effects of Methylpenicinoline (**1**) on the Production of Pro-Inflammatory Mediators and Cytokines in BV2 Microglia Stimulated with LPS

**Figure 4:**
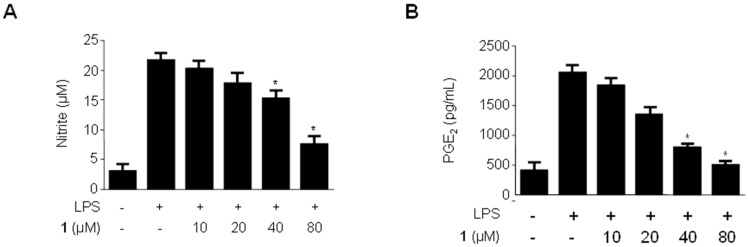

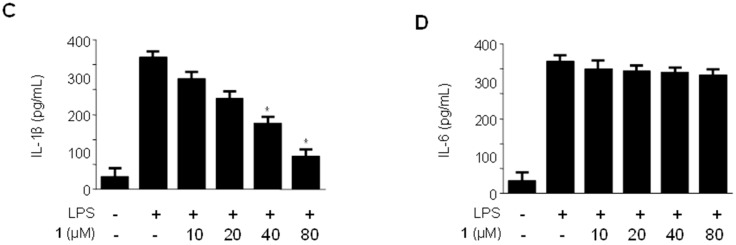
Effects of methylpenicinoline (**1**) on nitrite (**A**); PGE_2_ (**B**); IL-1β (**C**); and IL-6 (**D**) production in BV2 microglia stimulated with LPS. Cells were pre-treated for 30 min with the indicated concentrations of **1**, and then stimulated for 24 h with LPS (500 ng/mL). The concentrations of nitrite (A); PGE_2_ (B); IL-1β (C); and IL-6 (D) were determined as described in Section 3.5. Data represent the mean values of three experiments ± S.D. * *p* < 0.05 compared to the group treated with LPS alone.

We also evaluated the anti-inflammatory effects of methylpenicinoline (**1**) on LPS-stimulated BV2 microglia [25,26]. As shown in Figure 4, LPS treatment triggered an approximately 10-fold increase in nitrite concentration in the culture media. Pre-treatment of the microglia with compound **1** for 30 min decreased the production of NO in a concentration-dependent manner, with an IC_50_ value of 49 μM (Figure 4A). Under the same conditions, compound **1** also suppressed PGE_2_ production in a concentration-dependent manner, with an IC_50_ value of 34 μM (Figure 4B). In addition, compound **1** reduced IL-1β production in a concentration-dependent manner, with an IC_50_ value of 34 μM, but it did not affect the production of IL-6 (Figure 4D).

**Figure 5:**
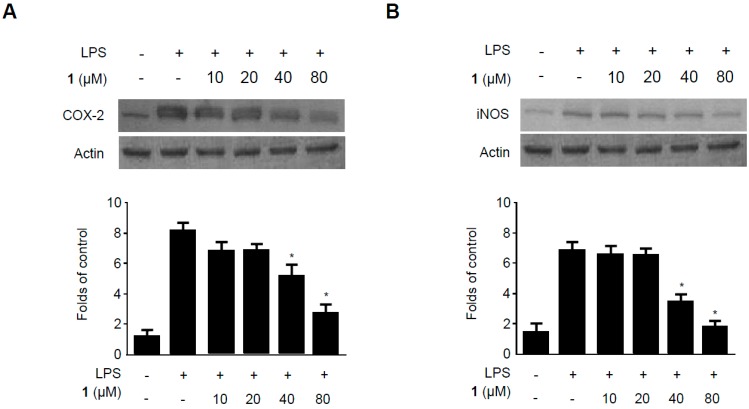
Effects of methylpenicinoline (**1**) on COX-2 (**A**) and iNOS (**B**) expression in RAW264.7 macrophages stimulated with LPS. Cells were pre-treated for 30 min with indicated concentrations of **1** and then for 24 h with LPS (500 ng/mL). Western blot analyses (A,B) were performed as described in described in Section 3.7; representative blots of three independent experiments are shown. Data are the mean values of three experiments ± S.D. * *p* < 0.05 compared to the group treated with LPS alone.

### 2.5. Effects of Methylpenicinoline (**1**) on the Expression of Pro-Inflammatory Enzymes in RAW264.7 Macrophages and BV2 Microglia Stimulated with LPS

It has been reported that LPS induces Toll-like receptor 4 expression and increases NO production by increasing the expression of iNOS [27]. As mentioned above, COX-2 is an inducible enzyme that catalyzes the production of prostaglandins [28,29]. The effects of compound **1** on LPS-induced iNOS and COX-2 expression have been evaluated by western blots (Figure 5). RAW264.7 macrophages were challenged with LPS (500 ng/mL) in the presence or absence of **1** at non-cytotoxic concentrations ranging from 10 to 80 µM. Western blotting revealed that compound **1** suppressed LPS-induced COX-2 (Figure 5A) and iNOS (Figure 5B) expression in a concentration-dependent manner. These results suggest that compound **1** inhibits pro-inflammatory cytokines and mediators by reducing iNOS and COX-2 protein expression.

**Figure 6:**
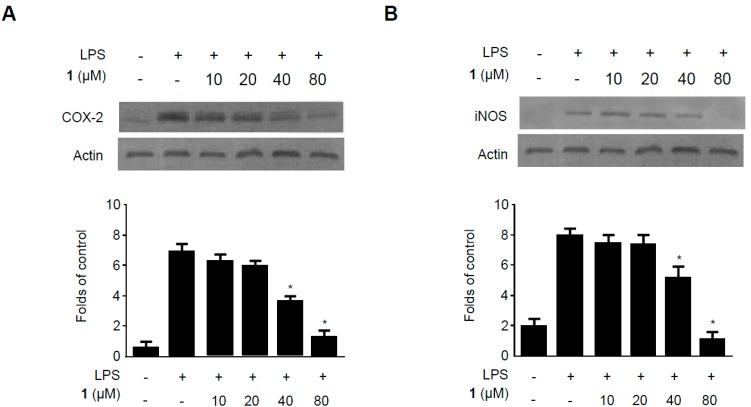
Effects of methylpenicinoline (**1**) on COX-2 (**A**) and iNOS (**B**) expression in BV2 microglia stimulated with LPS. Cells were pre-treated for 30 min with indicated concentrations of **1** and then for 24 h with LPS (500 ng/mL). Western blot analyses (A,B) were performed as described in Section 3.7; representative blots of three independent experiments are shown. Data are the mean values of three experiments ± S.D. * *p* < 0.05 compared to the group treated with LPS alone.

The effects of compound **1** on LPS-induced iNOS and COX-2 expression in BV2 microglia were also evaluated by western blot (Figure 6). BV2 microglia were challenged with LPS (500 ng/mL) in the presence or absence of **1** at non-cytotoxic concentrations ranging from 10 to 80 µM. Western blotting analysis revealed that compound **1** suppressed both LPS-induced COX-2 (Figure 6A) and iNOS (Figure 6B) expression in a concentration-dependent manner. These results suggest that compound **1** inhibits the expression of pro-inflammatory cytokines and mediators by reducing iNOS and COX-2 protein levels.

### 2.6. Effects of Methylpenicinoline (**1**) on NF-κB Activation in RAW264.7 Macrophages and BV2 Microglia Stimulated with LPS

**Figure 7:**
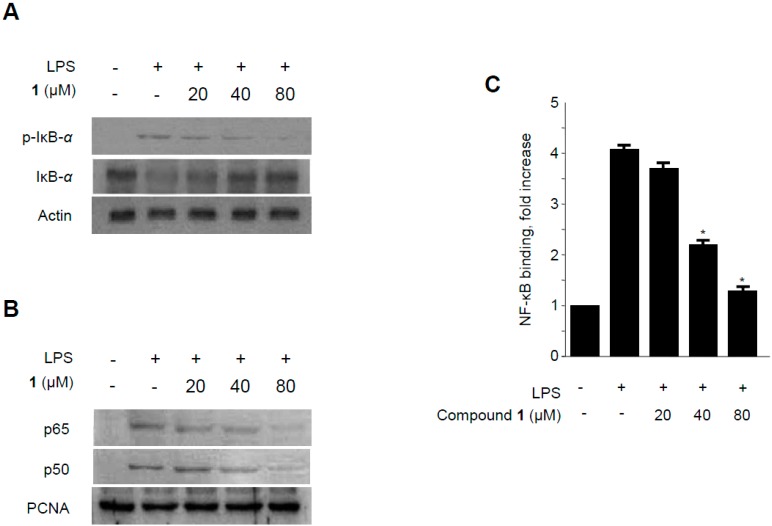
Effects of methylpenicinoline (**1**) on LPS-induced NF-κB activation in RAW264.7 macrophages. (**A**) Following pretreatment with **1** (20, 40, and 80 μM) for 30 min, cells were treated with LPS for 30 min. Total protein was isolated and western blot analysis was performed using specific IκB-α and p-IκB-α antibodies; (**B**) Nuclear extracts were prepared for a western blot of p65 and p50 of NF-κB, using specific anti-p65 and anti-p50 monoclonal antibodies; (**C**) A commercially available NF-κB ELISA (Active Motif) kit was used to test the nuclear extracts and determine the degree of NF-κB binding. The data shown are the mean values of three independent experiments ± S.D. * *p* < 0.05 compared to the group treated with LPS alone.

**Figure 8:**
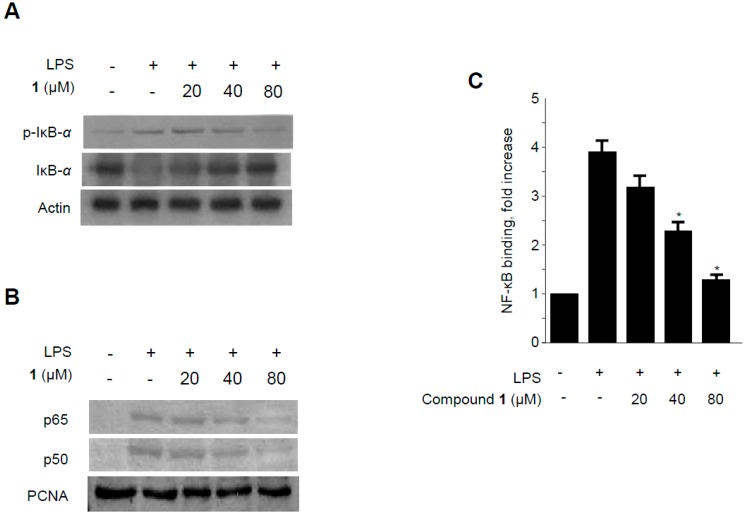
Effects of methylpenicinoline (**1**) on LPS-induced NF-κB activation in *BV2* microglia. (**A**) Following pretreatment with **1** (20, 40, and 80 μM) for 30 min, cells were treated with LPS for 30 min. Total proteins were prepared and western blot analysis was performed using specific IκB-α and p-IκB-α antibodies; (**B**) Nuclear extracts were prepared for western blot of p65 and p50 of NF-κB, using specific anti-p65 and anti-p50 monoclonal antibodies; (**C**) A commercially available NF-κB ELISA (Active Motif) kit was used to test the nuclear extracts and determine the degree of NF-κB binding. The data shown are the mean values of three independent experiments ± S.D. * *p* < 0.05 compared to the group treated with LPS alone.

NF-κB is a major transcription factor that has been shown to be essential for the expression of iNOS, COX-2, and inflammatory cytokines [30]. NF-κB is composed of two subunits (p65 and p50) which are present in the cytosol as inactive heterodimers bound to the inhibitory protein IκB-α [31]. Upon stimulation by pro-inflammatory signals, including LPS, IκB-α is phosphorylated by IκB kinase and proteolytically degraded via a 26S proteasome-mediated pathway that facilitates NF-κB translocation into the nucleus and thus regulates target gene transcription [32]. Therefore, the phosphorylation and degradation of IκB-α was estimated to investigate the mechanisms by which methylpenicinoline (**1**) suppresses the pro-inflammatory enzymes and mediators induced by LPS. As shown in Figure 7A, IκB-α in macrophages was phosphorylated and degraded after LPS treatment (500 ng/mL for 1 h). However, pre-treatment with **1** for 30 min, at concentrations ranging from 20 to 80 μM, significantly suppressed LPS-induced phosphorylation and degradation of IκB-α, thereby blocking NF-κB (p50 and p65) translocation into the nucleus. The protein expression of nuclear p50 and p65 increased after treatment with LPS for 1 h. However, this response was gradually inhibited by the treatment with **1** in a concentration-dependent manner (Figure 7B). Furthermore, to determine whether the decrease in iNOS and COX-2 gene expression after treatment with **1** was associated with the inhibition of the binding activity of NF-κB, RAW264.7 cells were treated with 20, 40, or 80 μM of **1** for 30 min, and then the nuclear extracts were prepared and analyzed using a Trans-AM NF-κB binding assay (Active Motif). Macrophages treated with LPS for 30 min showed an approximately four-fold higher NF-κB DNA-binding activity as compared to controls. In BV2 microglia*,* analogous effects of **1** on NF-κB activation were observed (Figure 8). These findings indicate that the anti-inflammatory effect of **1** is associated, at least in part, with the suppression of NF-κB activation.

### 2.7. Effects of Methylpenicinoline (**1**) on the Phosphorylation of MAPKs in RAW264.7 Macrophages and BV2 Microglia Stimulated with LPS

**Figure 9:**
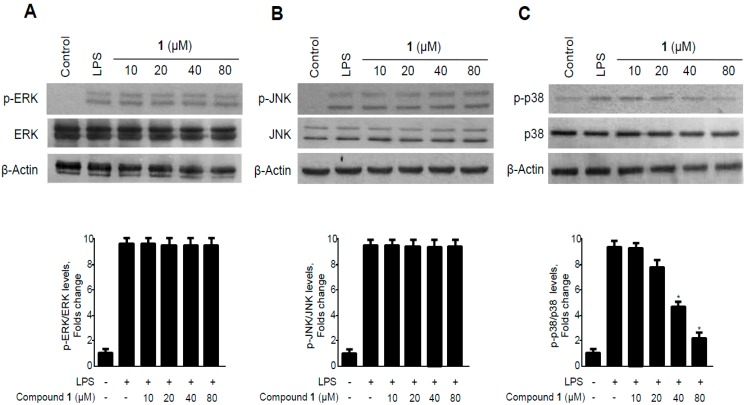
Effects of methylpenicinoline (**1**) on ERK, JNK, and p38 MAPK phosphorylation and protein expression in RAW264.7 macrophages. Cells were pre-treated for 30 min with the indicated concentrations of **1** and stimulated for 30 min with LPS (500 ng/mL) (**A**–**C**). The levels of (A) phosphorylated-ERK (p-ERK); (B) phosphorylated-JNK (p-JNK); and (C) phosphorylated-p38 MAPK (p-p38 MAPK) were determined by western blotting. Representative blots from three independent experiments are shown. Data represent the mean of three experiments ± S.D. * *p* < 0.05 compared to the group treated with LPS alone.

MAPK pathways are among the important signaling pathways that control the synthesis and release of pro-inflammatory mediators by activated macrophages during the inflammatory response [33]. The various MAPK family proteins, particularly ERK, JNK, and p38, are regarded as important targets for the development of anti-inflammatory agents [34]. To investigate whether methyl-penicinoline (**1**) suppressed inflammatory reactions mediated by the MAPK pathway, we assessed the effect of **1** on the LPS-induced phosphorylation of ERK, JNK, and p38 in RAW264.7 macrophages. As shown in Figure 9, the phosphorylation levels of ERK, JNK, and p38 increased after 30 min of treatment with LPS. However, pre-treatment with methyl-penicinoline (**1**) for 30 min, at 10 to 80 μM, significantly inhibited the LPS-induced phosphorylation of JNK in a concentration-dependent manner (Figure 9B), while ERK and p38 phosphorylation was not affected. On the other hand, neither LPS nor **1** affected the expression level of ERK, JNK, and p38. These data suggest that methylpenicinoline (**1**) regulates inflammatory reactions by inhibiting the MAPK-JNK signaling pathway.

**Figure 10:**
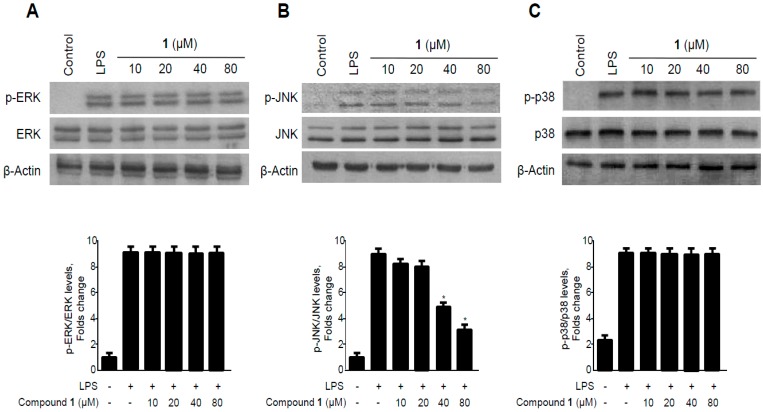
Effects of methylpenicinoline (**1**) on ERK, JNK, and p38 MAPK phosphorylation and protein expression in BV2 microglia. Cells were pre-treated for 30 min with the indicated concentrations of **1** and stimulated for 30 min with LPS (500 ng/mL) (**A**–**C**). The levels of (A) phosphorylated-ERK (p-ERK); (B) phosphorylated-JNK (p-JNK); and (C) phosphorylated-p38 MAPK (p-p38 MAPK) were determined by western blotting. Representative blots from three independent experiments are shown. Data represent the mean values of three experiments ± S.D. * *p* < 0.05 compared to the group treated with LPS alone.

In BV2 microglia, pre-treatment with methylpenicinoline (**1**) for 30 min, at 10 to 80 μM, significantly inhibited the LPS-induced phosphorylation of p38 in a dose-dependent manner (Figure 10C), while ERK and JNK phosphorylation was not affected. On the other hand, neither LPS nor **1** affected the expressions of ERK, JNK, and p38. These data suggest that methylpenicinoline (**1**) regulates inflammatory reactions by inhibiting p38 MAPK signaling pathways.

## 3. Experimental Section

### 3.1. General

NMR spectra (1D and 2D) were recorded in DMSO-*d*_6_ with a JEOL JNM ECP-400 spectrometer operating at 400 MHz for ^1^H and at 100 MHz for ^13^C, and chemical shifts were referenced relative to the corresponding residual solvent signals (δ_H_/δ_C_ = 2.50/39.5). HSQC and HMBC experiments were optimized for ^1^*J*_CH_ = 140 Hz and ^n^*J*_CH_ = 8 Hz, respectively. Flash column chromatography was performed using octadecyl-functionalized silica gel C_18_ (12 nm, S-75 μm, YMC, Japan) and silica gel 60 (230–400 mesh, Merck, Darmstadt, Germany). TLC was carried out on silica gel 60 F_254_ plates (Merck). Medium-pressure liquid chromatography (MPLC) was conducted with a Yamazen MPLC system and an Ultra Pak SI-40A silica gel column (11 × 300 mm, Yamazen, Osaka, Japan). Dulbecco’s modified Eagle’s medium (DMEM), fetal bovine serum (FBS), and other tissue culture reagents were purchased from Gibco BRL Co. (Grand Island, NY, USA). All chemicals were obtained from Sigma Chemical Co. (St. Louis, MO, USA). Primary antibodies, including anti-COX-2, iNOS, IкB-α, p-IкB-α, p50, p65, as well as anti-mouse, goat, and rabbit secondary antibodies were purchased from Santa Cruz Biotechnology (Santa Cruz, CA, USA). p-ERK, ERK, p-JNK, JNK, p-p38, and p38 antibodies were obtained from Cell Signaling Technology (Danvers, MA, USA). Enzyme-linked immunosorbent assay (ELISA) kits for PGE_2_, IL-1β, and IL-6 were purchased from R & D Systems, Inc. (Minneapolis, MN, USA).

### 3.2. Specimen Collection and Identification of the Marine-Derived Fungus Penicillium *sp.* SF-5995

*Penicillium* sp. SF-5995 (deposited at the College of Medical and Life Sciences fungal strain repository, Silla University) was isolated from an unidentified soft coral that was manually collected using scuba equipment at a depth of 4.5–21 m at Terra Nova bay (74, 37'39.895"S, 164, 14'26.895"E), Antarctica, in January 2012. The sample was stored in a sterile plastic bag and transported to the laboratory, where it was kept frozen until further processing. One gram of the sample was ground with a mortar and pestle, and it was mixed with sterile seawater (10 mL). A portion (0.1 mL) of the sample was processed utilizing the spread plate method in potato dextrose agar (PDA) medium containing seawater. The plate was incubated at 25 °C for 14 d. After purifying the isolates several times, the final pure culture was maintained as a glycerol suspension (20%, w/v) at −70 °C. This fungus was identified based on the analysis of the ribosomal RNA (rRNA) sequence. A GenBank search with the 28S rRNA gene of SF-5995 (GenBank accession number KF562347) indicated *Penicillium steckii* (HM469415), *Penicillium chrysogenum* (FJ890400), and *Penicillium paxilli* (FJ890402), as the closest matches, showing sequence identities of 99.63%, 98.63%, and 98.01%, respectively. Therefore, the marine-derived fungal strain SF-5995 was characterized as a *Penicillium* sp., but was not definitively identified at the species level.

### 3.3. Fermentation, Extraction, and Isolation of Methylpenicinoline (**1**) from Penicillium *sp.* SF-5995

The fungal strain was cultured on 50 Petri-dishes (90 mm), each containing 20 mL of potato dextrose agar media [0.4% (*w*/*v*) potato starch, 2% (*w*/*v*) dextrose, 3% (*w*/*v*) NaCl, 1.5% (*w*/*v*) agar]. Plates were individually inoculated with 2 mL seed cultures of the fungal strain. Plated cultures were incubated at 25 °C for a period of 14 d. Extraction of the agar media with EtOAc (4 × 1000 mL) provided an organic phase, which was then concentrated *in vacuo* to yield 474.3 mg of extract. The EtOAc extract was subjected to C_18_-functionalized silica gel open column chromatography and eluted with a stepwise gradient of 20%, 40%, 60%, 80%, and 100% (*v*/*v*) of MeOH in H_2_O (500 mL each). The fraction (32.1 mg) eluted with 60% MeOH was subjected to HPLC using a reversed phase with a gradient from 40% to 60% MeOH in H_2_O over 50 min to obtain compound **1** [2.0 mg, *t*_R_ = 31.3 min]. ^1^H-NMR (400 MHz, DMSO-*d*_6_) δ: 11.80 (1H, s, 7-NH), 8.06 (1H, d, *J* = 8.0 Hz, H-12), 7.69 (2H, br d, H-9, H-10), 7.35 (1H, dt, *J* = 8.0 Hz, 4.0 Hz, H-11), 7.13 (1H, br t, H-3), 6.46 (1H, br d, *J* = 3.9 Hz, H-1), 3.67 (3H, s, H-17); ^13^C-NMR (100 MHz, DMSO-*d*_6_) δ: 174.1 (C-14), 168.3 (C-16), 141.2 (C-6), 140.3 (C-8), 132.8 (C-10), 125.3 (C-12), 124.8 (C-13), 124.1 (C-11), 123.6 (C-5), 123.1 (C-3), 119.2 (C-9), 114.0 (C-14), 112.4 (C-1), 110.4 (C-2), 52.5 (C-17).

### 3.4. Cell Culture and Viability Assay

RAW264.7 macrophages and BV2 microglia were maintained at a density of 5 × 10^5^ cells/mL in DMEM medium supplemented with 10% heat-inactivated FBS, penicillin G (100 units/mL), streptomycin (100 mg/mL), and L-glutamine (2 mM), and were incubated at 37 °C in a humidified atmosphere containing 5% CO_2_. The effect of the various experimental treatments on cell viability were evaluated by determining mitochondrial reductase function with an assay based on the reduction of the tetrazolium salt 3-[4,5-dimethylthiazol-2-yl]-2,5-diphenyltetrazolium bromide (MTT) into formazan crystals [35]. The formation of formazan is proportional to the number of functional mitochondria in the living cells. For the determination of cell viability, cells were maintained at a 1 × 10^5^ cells/mL in each well of the 96-well plates. After incubated for 6 h, cells were treated for 24 h with the indicated concentrations of **1** (5–160 μM). And then, 50 μL of MTT (2.5 mg/mL) was added to the each well in a fresh medium at a final concentration of 0.5 mg/mL, and the mixture was further incubated for 3–4 h at 37 °C, and the liquid was removed from the wells in turn. Thereafter, the formazan formed was dissolved in 150 μL of acidic 2-propanol, and the optical density was measured at 590 nm. The optical density of the formazan formed in the control (untreated) cells was considered to indicate 100% viability.

### 3.5. Determination of the Production of Nitrite, PGE_2_, IL-6, and IL-1β

The production of nitrite, a stable end product of NO oxidation, was used as a measure of iNOS activity. The nitrite present in the conditioned medium was determined using a method based on the Griess reaction [36]. The concentrations of PGE_2_, IL-6, and IL-1β in the culture medium were determined using ELISA kits (R&D Systems) according to the manufacturer’s instructions.

### 3.6. Preparation of Cytosolic and Nuclear Fractions

RAW264.7 macrophages and BV2 cells were homogenized in PER-Mammalian Protein Extraction Buffer (1:20, w:v) (Pierce Biotechnology, Rockford, IL, USA) containing freshly added protease inhibitor cocktail I (EMD Biosciences, San Diego, CA, USA) and 1 mM PMSF. The cytosolic fraction of the cells was prepared by centrifugation at 15,000× *g* for 10 min at 4 °C. Nuclear and cytoplasmic extracts of cells were prepared using NE-PER nuclear and cytoplasmic extraction reagents (Pierce Biotechnology), respectively.

### 3.7. Western Blot Analysis

RAW264.7 macrophages and BV2 cells were harvested and pelleted by centrifugation at 200× *g* for 3 min. Then, the cells were washed with PBS and lysed in 20 mM Tris-HCl buffer (pH 7.4) containing a protease inhibitor mixture (0.1 mM phenylmethanesulfonyl fluoride, 5 mg/mL aprotinin, 5 mg/mL pepstatin A, and 1 mg/mL chymostatin). Protein concentration was determined using a Lowry protein assay kit (P5626; Sigma). Thirty micrograms of protein from each sample was resolved by 12% sodium dodecyl sulfate polyacrylamide gel electrophoresis (SDS-PAGE), and then electrophoretically transferred onto a Hybond enhanced chemiluminescence (ECL) nitrocellulose membrane (Bio-Rad, Hercules, CA, USA). The membrane was blocked with 5% skim milk and sequentially incubated with the primary antibody (Santa Cruz Biotechnology and Cell Signaling Technology) and a horseradish peroxidase-conjugated secondary antibody followed by ECL detection (Amersham Pharmacia Biotech, Piscataway, NJ, USA).

### 3.8. DNA Binding Activity of NF-κB

The DNA-binding activity of NF-κB in nuclear extracts was measured using the TransAM kit (Active Motif, Carlsbad, CA, USA) according to the manufacturer’s instructions. Briefly, 30 μL of complete binding buffer (DTT, herring sperm DNA, and binding buffer AM3) was added to each well. Nuclear extracts from RAW264.7 macrophages and BV2 cells were stimulated for 30 min with LPS and treated with different-concentrations of methyl-penicinoline (**1**); these were used as the samples. Then, 20 μL of the samples in complete lysis buffer was added to each well (20 μg of nuclear extract diluted in complete lysis buffer). The plates were incubated for 1 h at room temperature with mild agitation (100 rpm on a rocking platform). After washing each well with wash buffer, 100 μL of diluted NF-κB antibody (1:1000 dilution in 1× antibody binding buffer) was added to each well, and then the plates were incubated further for 1 h as before. After washing each well with wash buffer, 100 μL of diluted HRP-conjugated antibody (1:1000 dilution in 1× antibody binding buffer) was added to each well, followed by 1 h incubation as before. One hundred microliters of developing solution was added to each well for 5 min, followed by the addition of stop solution. Finally, the absorbance of each sample at 450 nm was determined with a spectrophotometer within 5 min.

### 3.9. Statistical Analysis

Data was expressed as the mean ± S.D. of at least three independent experiments. To compare three or more groups, one-way analysis of variance followed by the Newman-Keuls post hoc test was used. Statistical analysis was performed with GraphPad Prism software, version 3.03 (GraphPad Software Inc., San Diego, CA, USA).

## 4. Conclusions

Chemical investigation of the marine-derived fungus *Penicillium* sp. SF-5292 provided a pyrrolyl 4-quinoline alkaloid, methylpenicinoline (**1**), as an anti-inflammatory constituent. Methyl-penicinoline (**1**) has been previously isolated from the marine-derived fungus *Penicillium* sp. ghq208 and has a moderate cytotoxicity on HepG2 cells [37]. This alkaloid has also been isolated from the fungus *Auxarthron reticulatum* and has a weak antagonist activity at cannabinoid CB_1_ and CB_2_ receptors [17]. However, the effects of methylpenicinoline (**1**) on activated macrophages and microglia have not been elucidated. 

In the present study, methylpenicinoline (**1**) inhibited the production of the pro-inflammatory mediators, PGE2 and NO (Figure 3 and Figure 4), and these inhibitory effects were mediated through down-regulation of COX-2 and iNOS protein expression (Figure 5 and Figure 6) in LPS-stimulated RAW267 macrophages. To explore the possible underlying mechanism, two major signaling pathways (*i.e.*, NF-κB and MAPK pathways) were examined. Activation of the NF-κB protein plays a pivotal role in inflammation due to its ability to induce transcription of pro-inflammatory genes. We found that down-regulation of pro-inflammatory mediators by methylpenicinoline (**1**) is due, at least in part, to inhibition of the NF-κB pathway in LPS-stimulated RAW267 macrophages and BV2 microglia. LPS also activates MAPK family members and the MAPK signaling pathway also plays a crucial role in inflammatory mediator induction [38]. Therefore, further experiments were performed to elucidate the effects of methylpenicinoline (**1**) on the activation (phosphorylation) of MAPKs. Our results indicate that methylpenicinoline (**1**) suppresses the phosphorylation of JNK in LPS-stimulated RAW264.7 cells, but activation of ERK and p38 were not altered by **1**. On the other hand, methylpenicinoline (**1**) suppressed only the activation of p38 in BV2 microglia. These data suggests that the anti-inflammatory effects of **1** were also due to inhibition of the MAPKs signaling pathway, although the effects of **1** are somewhat different between cell types. 

In summary, the results demonstrate that methylpenicinoline (**1**) exerts anti-inflammatory effects by suppressing LPS-induced expression of pro-inflammatory mediators through the NF-κB and MAPK pathways in RAW264.7 cells and BV2 microglia. Therefore, methylpenicinoline (**1**) may be a potential chemotherapeutic candidate for the management of inflammatory and neurodegenerative disorders.

## Supplementary Materials

Supplementary materials can be accessed at: http://www.mdpi.com/1420-3049/19/11/18073/s1.

## Supplementary Files

Supplementary File 1
