# Supplementary material for: Anti-Inflammatory Effect of Methylpenicinoline from a Marine Isolate of Penicillium sp. (SF-5995): Inhibition of NF-κB and MAPK Pathways in Lipopolysaccharide-Induced RAW264.7 Macrophages and BV2 Microglia
Source: Molecules. 2014 Nov 5;19(11):18073–89. doi: 10.3390/molecules191118073 (PMC6271136; doi:10.3390/molecules191118073)

Supplementary Materials

Figure S1. ^{1}H-NMR spectrum of compound **1** at 400 MHz in CD_3OD-*d*$_6$.

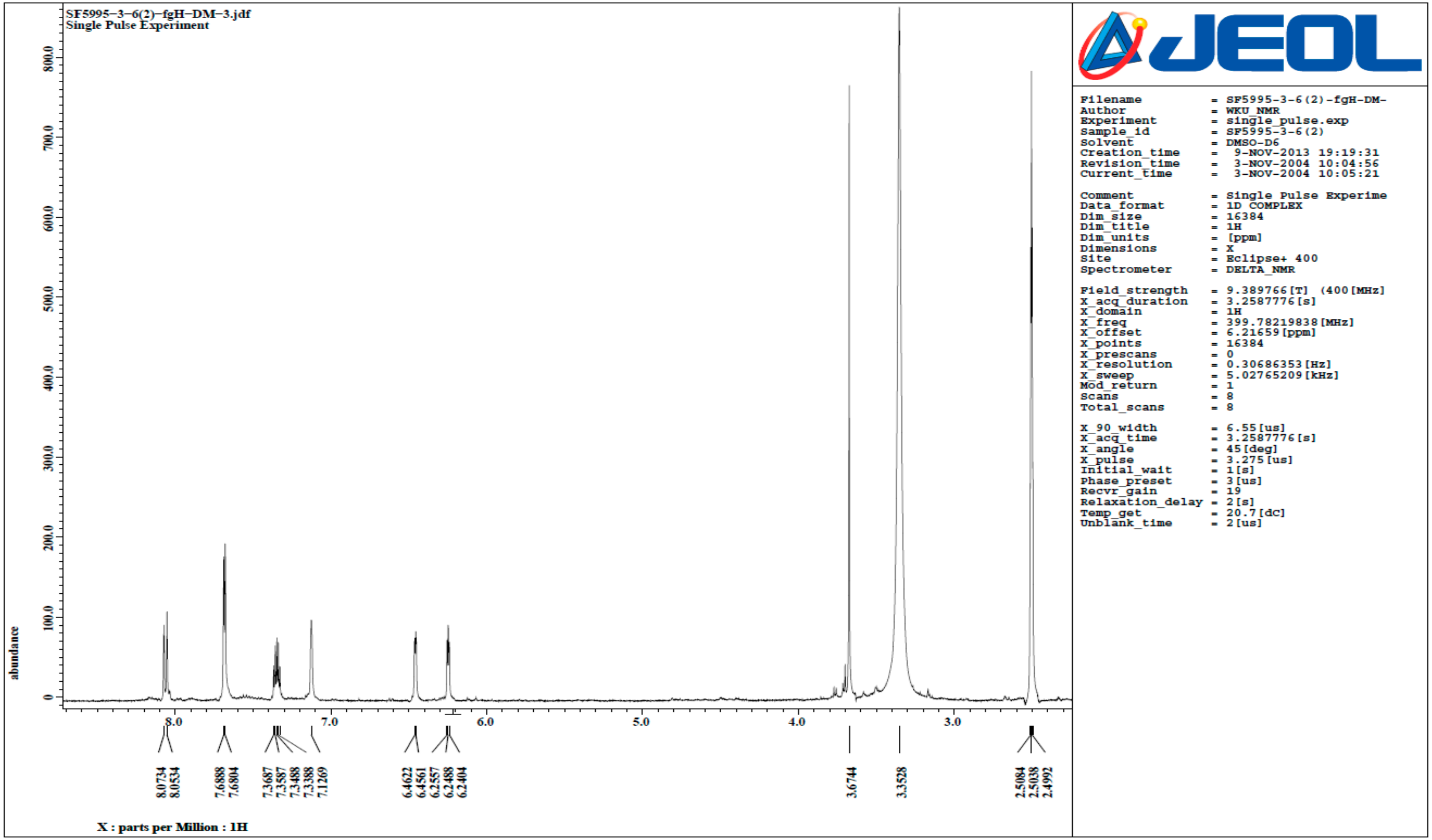

Figure S2. ^{13}C-NMR spectrum of compound **1** at 100 MHz in CD_3OD-*d*$_6$.

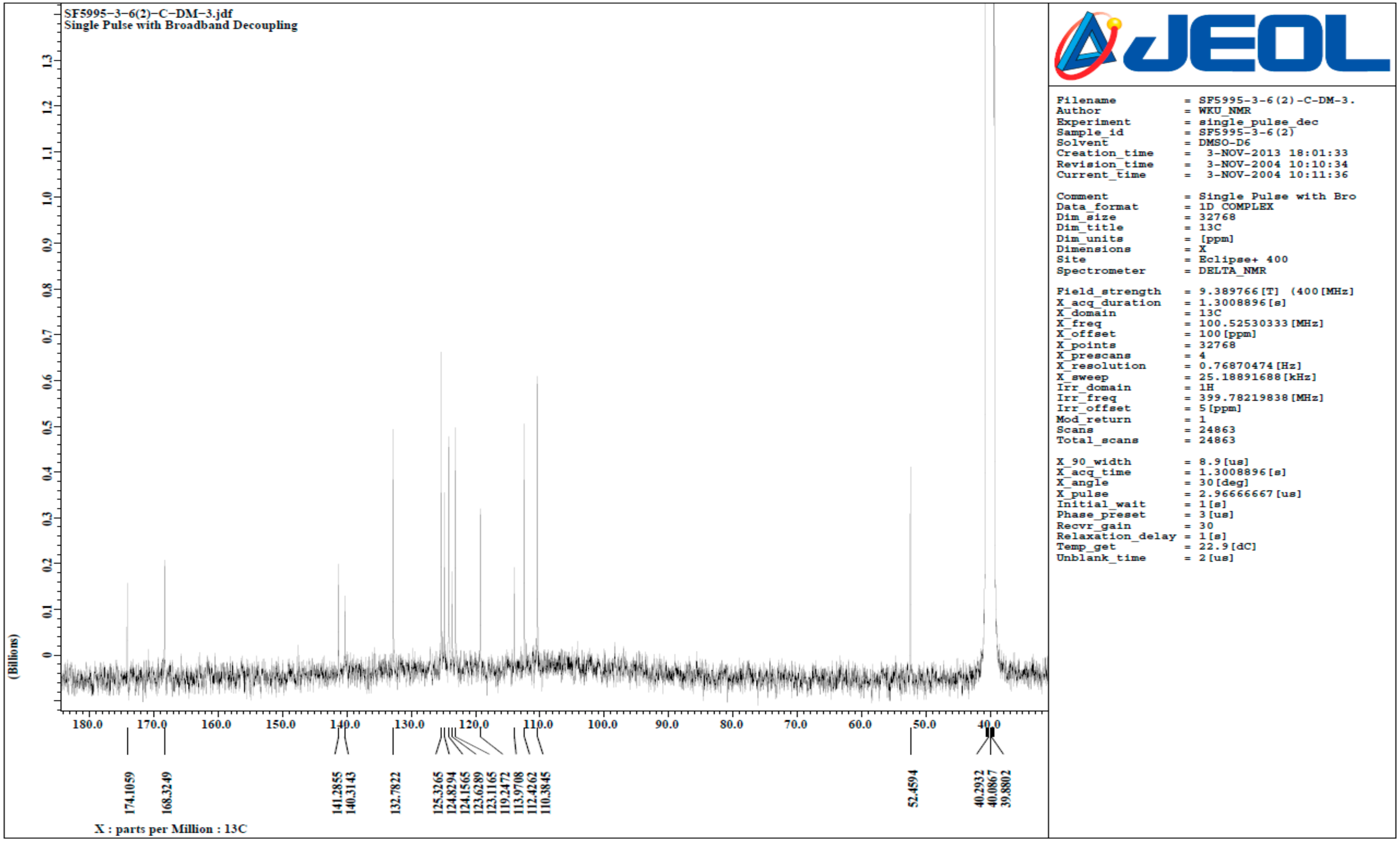

Figure S3. HR-ESIMS of compound **1**.

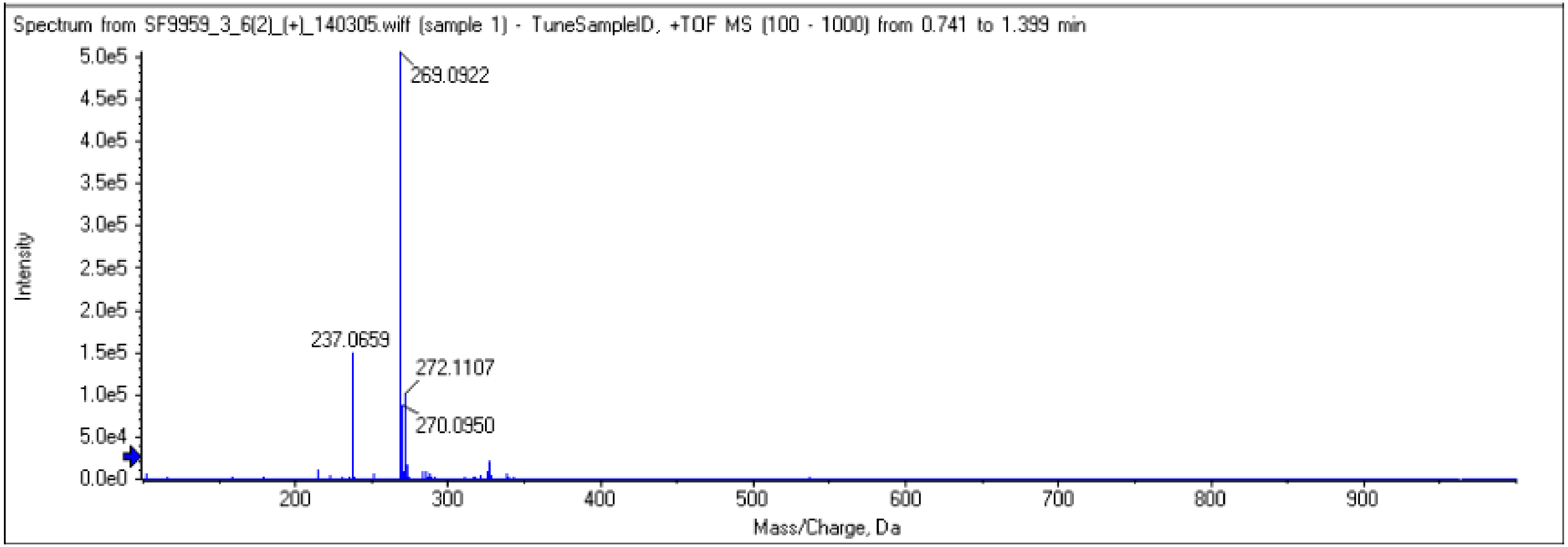

Supplement: Supplementary File 1 [file molecules-19-18073-s001.pdf]
